# Development and Properties of Polymeric Nanocomposite Coatings

**DOI:** 10.3390/polym11050852

**Published:** 2019-05-10

**Authors:** Muddasir Nawaz, Noor Yusuf, Sehrish Habib, Rana Abdul Shakoor, Fareeha Ubaid, Zubair Ahmad, Ramazan Kahraman, Said Mansour, Wei Gao

**Affiliations:** 1 Center of Advanced Materials (CAM), Qatar University, 2713 Doha, Qatar; bie.mudasir@gmail.com (M.N.); noorsyusuf@hotmail.com (N.Y.); sehrish.habib@qu.edu.qa (S.H.); fareeha.ubaid@qu.edu.qa (F.U.); Zubairtarar@qu.edu.qa (Z.A.); 2 Department of Chemical Engineering, Qatar University, 2713 Doha, Qatar; ramazank@qu.edu.qa; 3 Qatar Energy and Environment Research Institute, Hamad Bin Khalifa University, Qatar Foundation, 34110 Doha, Qatar; smansour@hbku.edu.qa; 4 Department of Chemical and Materials Engineering, The University of Auckland, Private Bag 92019, Auckland 1142, New Zealand; w.gao@auckland.ac.nz

**Keywords:** polymer, nanocontainers, nanocomposite coatings, self-healing, inhibitor, corrosion

## Abstract

Polymeric-based nanocomposite coatings were synthesized by reinforcing epoxy matrix with titanium nanotubes (TNTs) loaded with dodecylamine (DOC). The performance of the developed nanocomposite coatings was investigated in corrosive environments to evaluate their anti-corrosion properties. The SEM/TEM, TGA, and FTIR analysis confirm the loading of the DOC into the TNTs. The UV-Vis spectroscopic analysis confirms the self-release of the inhibitor (DOC) in response to the pH change. The electrochemical impedance spectroscopic (EIS) analysis indicates that the synthesized nanocomposite coatings demonstrate superior anticorrosion properties at pH 2 as compared to pH 5. The improved anticorrosion properties of nanocomposite coatings at pH 2 can be attributed to the more effective release of the DOC from the nanocontainers. The superior performance makes polymeric nanocomposite coatings suitable for many industrial applications.

## 1. Introduction

Carbon steel pipelines are used widely in the oil and gas industry for the transportation of liquids and gases. However, carbon steel pipelines suffer from poor corrosion resistance in solutions containing aggressive species like chloride ions [1,2,3]. Localized corrosion of pipelines in the oil and gas industry has been identified as a major factor causing pipeline failure worldwide, leading to mechanical failure and economic loss [4]. Localized corrosion includes pitting on carbon steel surface, which is harder to detect than uniform corrosion. Owing to the deleterious effect of corrosion, different techniques have been employed to improve the corrosion resistance of carbon steel [5,6,7]. Towards this goal, the corrosion resistance of steel has been improved by incorporation of insoluble, hard ceramic particles such as carbides and nitrides into steel [8]. On the other hand, corrosion mitigation using various types of corrosion inhibitors and coatings (organic and metallic) has also been conducted [9,10]. Organic inhibitors containing nitrogen and acetylenic alcohols have demonstrated promising efficiency [11,12,13]. Among various organic corrosion inhibitors, the dodecylamine (DOC) has been widely studied in aggressive acidic media [14,15]

Corrosion protection of metal surfaces by applying polymeric coatings is reliable and effective. Polymeric coatings provide a dense barrier for water and corrosive species from penetrating to the metal surface [16,17,18]. Epoxy resins are used for coating metals due to its strong chemical resistance and adhesion to metal surfaces [19]. However, when the epoxy-based barrier is damaged or scratched, the aggressive agents diffuse into the metal surface and cause corrosion [5]. Therefore, for control and delay, active corrosion suitable corrosion inhibitors are introduced into the epoxy coating [20]. Chromate containing coatings have been used for quite a time in the industry for the anticorrosion purpose. However, strict environmental and health regulations have been established due to the toxicity and cancer genic effects of chromates [20,21]. Furthermore, direct doping of organic corrosion inhibitors into the epoxy reduces the inhibition efficiency [20]. The decrease in inhibition efficiency is often noticed due to undesirable reactions between the inhibitor molecules and epoxy. Consequently, the smart coatings where the corrosion inhibitor is encapsulated into the nanocontainers and reinforced into the matrix are developed for solving this problem. The nanocontainers sense the corrosive dynamics and release the inhibitor on a required basis. Smart coatings are sensitive to different corrosive environmental changes such as pH change, temperature, mechanical damage, etc., and release the corrosion inhibitor when needed to mitigate corrosion [22]. Smart coatings are economically and environmentally friendly since the inhibitor is only released on a required basis. The corrosion inhibitors stored inside the inorganic nanocarriers are necessary to attain more efficient and reliable self-healing coatings that has been reported in previous studies. [23,24,25]. Several types of corrosion inhibitors and self-healing agents can be deposited on the surface or loaded inside hollow or porous inorganic carriers.

The most reported potential nanocontainers to host corrosion inhibitors are titanium dioxide nanotubes (TNTs) [26,27], mesoporous silica nanotubes (MSNTs) [28], and halloysite nanotubes (HNTs) [29]. Yuanchao et al. [16] evaluated the corrosion resistance performance of benzotriazole as corrosion inhibitor by loading it inside hollow silica particles and corrosion inhibitor was released in response to pH changes in the electrolyte. Poornima et al. [29] used halloysite nanocontainers to encapsulate the epoxy monomer and studied the self-healing and corrosion behavior of the HNT’s doped epoxy coatings. Kinetics of releasing of corrosion inhibitors loaded within halloysite lumen was also studied by Lvov et al. [30]. Encapsulation and release of 8-hydroxyquinoline inside the TiO_2_ nanocontainers were studied by Ioannis et al. [31]. Falcon et al. [28] reported the release of dodecylamine inhibitor from mesoporous silica nanotubes. It was demonstrated that the release of dodecylamine (DOC) is sensitive to pH change. A faster release has been reported at pH 2.0 than at pH 6.5 and pH 9.0 [28]. Epoxy coatings are formulated with species, which enable them to hold back the water suction and capture the chlorides and carriers release the corrosion inhibitors. Nanocomposite coatings having different additives without detrimental effects on their barrier properties, and interactive combination of corrosion inhibitors are expressed in literature [32]. TNTs are attractive in the coating industry due to their affordability, high corrosion resistivity, and chemical stability [26,33]. TNTs have been synthesized using template-assisted method [34], anodizing of metal substrates [35,36], and the hydrothermal method [26]. However, the loading of TNTs with DOC and their performance evaluation under different corrosive conditions has not been studied yet.

In this work, we have synthesized TNTs using hydrothermal method [27], which were then loaded with DOC in a weight ratio of 5%. TNTs loaded with DOC were then thoroughly dispersed into the epoxy matrix and coated on the steel substrate to develop smart coatings. Prepared TNTs and the nanocomposite coatings were then characterized. It was observed that the self-release of DOC from the nanocontainers (TNTs) is sensitive to the pH of the corrosive medium. The synthesized nanocomposite coatings demonstrate better corrosion resistance at pH 2 than pH 5. The improved properties of nanocomposite coatings at pH 2 can be attributed to the more efficient release of the DOC from the nanocontainers. The decent corrosion resistance of the developed nanocomposite coatings makes them attractive for industrial applications.

## 2. Materials and Methods

### 2.1. Materials

Titanium (IV) dioxide (TiO_2_) also known as titania, hydrochloric acid (37%), sodium hydroxide pallets, and ethanol were purchased from Sigma Aldrich, Darmstadt, Germany. Dodecyl amine (DOC, 98% pure) was purchased from Alfa-Aesar, Ward Hill, MA, United States. Epoxy resin (815C) and EPIKURE as its curing agent were purchased from Sigma Aldrich, Darmstadt, Germany. Plates of plain carbon steel (30L × 30W × 1.0T) mm^3^ used as substrates were locally purchased. The substrates were polished with silicon carbide (SiC) emery papers from 120 to 800 grit size and then rinsed thoroughly with distilled water.

### 2.2. Synthesis of TiO_2_ Nanotubes (TNTs)-Nanocontainers

Titanium oxide nanotubes (TNTs) or nanocontainers were synthesized using the hydrothermal method as reported in the literature. A total of 1.2 g of TiO_2_ powder was added into 20.0 ml of 10 M NaOH in Teflon beaker and stirred for 15 min. The mixture was then put into Teflon-lined autoclave and heated at 130 °C for 10 h in a preheated oven. Precipitates obtained after centrifugation were washed with distilled water, dipped into 0.1 M HCl solution for 30 min, and then washed again with distilled water until pH 7 was attained. Finally, the synthesized powder was dried at 80 °C for 3 h to obtain TNTs.

### 2.3. Loading of TNTs with an Inhibitor

**Figure 1:**
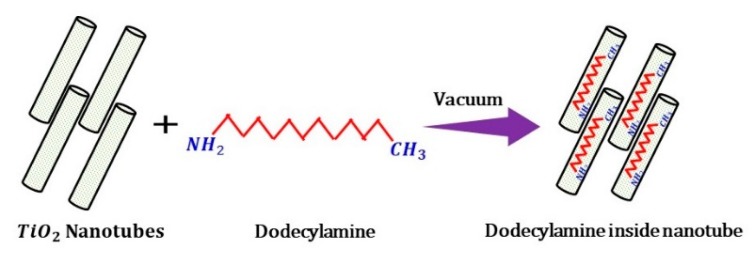
Schematic diagram showing the loading of dodecylamine (DOC) inside titanium oxide nanotubes (TNTs).

The synthesized TiO_2_ nanotubes (TNTs) were loaded with the corrosion inhibitor, dodecylamine (DOC), following the procedure adopted by Price et al. [37]. Then, 300 mg of synthesized TNTs were to be mixed into a DOC solution 25.0 mg/ml in ethanol. After sonication, the solution was kept inside the vacuum jar for 24 h to complete the loading process; during the loading process, the air trapped in the nanotubes was replaced with DOC. A proposed schematic illustration of this encapsulation process is presented in Figure 1.

### 2.4. Preparation of Nanocomposite Coatings

The nanocomposite coatings were developed in several steps; (i) 5.0 wt.% of DOC-loaded TNTs were thoroughly dispersed into the epoxy matrix resin, (ii) the addition of the hardener into the epoxy resin with the stoichiometric ratio of epoxy to the curing agent as 100:11, (iii) homogenization at room temperature, (iv) degassing of epoxy with sonication for 10 min, (v) application of processed epoxy mixture on steel substrate by doctor blade, and (vi) curing of the developed coatings at room temperature for 48 h.

### 2.5. Characterization

The morphological characterizations of synthesized TNTs and TNTs loaded with DOC were done by field emission scanning electron microscopy FE-SEM-Nova Nano-450 (FEI, New York, NY, USA) and transmission electron microscopy TEM TALOS F200X (FEI, New York, NY, USA). The structural and phase analysis of microcapsules was performed through X-ray diffraction analysis PANanalytical (Empyrean, Royston, United Kingdom) X’pert Pro Cu (Kα) with a scanning rate of 2°/min and 2θ from scanning angle ranging between 10° ≤ 90°. Fourier transform infrared spectroscopy (FTIR) was also performed to confirm the loading of DOC in TNTs. The Spectrum was recorded in the range of 4000–500 cm^−1^ in the transmission mode using the FTIR Frontier (PerkinElmer, Waltham, MA, USA) instrument. TGA synchronization analyzer TG4000 (PerkinElmer) was used to investigate the thermal stability of as-synthesized TNTs and TNTs loaded with DOC. Thermal stability was studied in the temperature range from room temperature to 600 °C with a heating rate of 20 °C/min. Self-release of the inhibitor encapsulated in TNTs was investigated by UV-vis spectroscopic analysis (LAMBDA 650 UV/Vis Spectrophotometer (PerkinElmer). The amount of the released DOC from the TNTs was measured as a function of time at pH 2 and 5. The charges on TNTs before and after loading with DOC were determined to employ zeta potential equipment Zeta sizer, Nano ZSP (Malvern, Westborough, MA, USA) to check the possibility of their agglomeration. Electrochemical impedance spectroscopy 30K BOOSTER Potentiostat/Galvanostat/ZRA (Gamry, Warminster, PA, USA) was carried out in 3.5 wt.% of NaCl solution at a pH level of 2 and 5 to evaluate the corrosion resistance of coatings. It was conducted within the frequency range of 0.1 to 100 KHz at OCP and the rms signal was 10 mv.

## 3. Results and Discussions

### 3.1. Morphological Analysis

**Figure 2:**
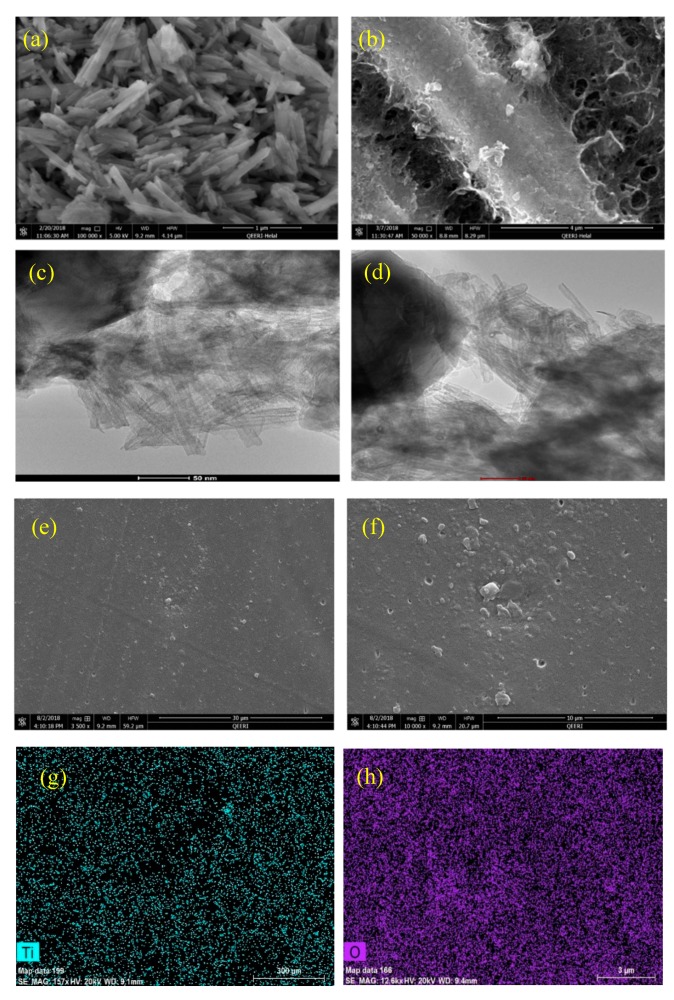
FE-SEM images: (**a**) As-synthesized TNTs, (**b**) TNTs loaded with DOC; HRTEM Images: (**c**) As-synthesized TNTs (**d**) TNTs loaded with DOC, (**e,f**) epoxy coating with 5 wt.% loaded nanocontainers (**g,h**) EDX elemental mapping showing the presence of Ti and O.

Figure 2 shows the (FE-SEM) and (HR-TEM) images of the as-synthesized TNTs and TNTs loaded with DOC. The tubular structure of as-synthesized TNTs is confirmed as shown in Figure 2a. The TNTs are relatively uniform in size and ordered in the stack. This result is in agreement with the results reported earlier in [38]. Figure 2b shows the sticking of DOC on the outer sides of TNTs in addition to the loading of DOC into the TNTs. The hollow nature of TNTs is visible in Figure 2c. However, the blackening of hollow TNTs after loading indicates successful loading of TNTs with DOC (Figure 2d). The SEM images of the synthesized coatings shown in Figure 2e,f indicates that the coating surface is relatively uniform and scratch free and does not contain any significant defects. EDX elemental mapping of epoxy coatings are shown in Figure 2g,h. It is clear from the elemental mapping that TNTs are properly mixed and homogenously distributed in the epoxy coating. The presence of Ti and O confirms the existence of TiO_2_ nanotubes. Furthermore, no presence of any foreign element or impurity in the EDX analysis confirming the high quality of the developed coatings.

### 3.2. Structural Analysis

**Figure 3:**
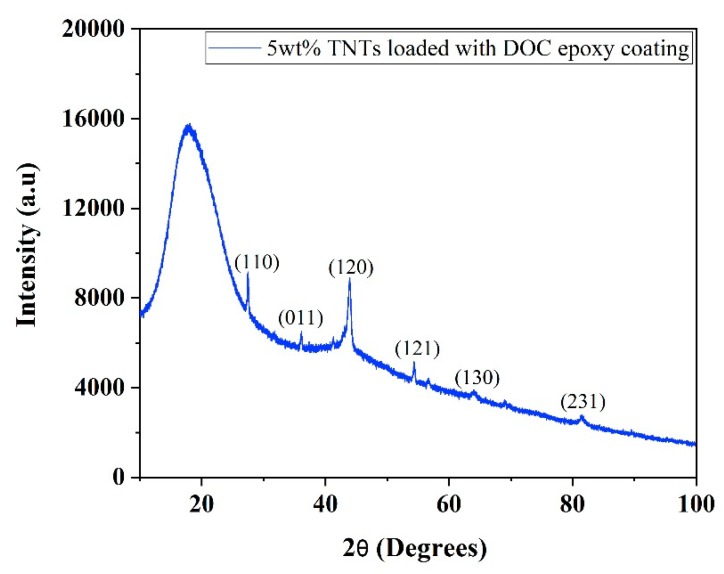
XRD pattern of as-synthesized nanocomposite epoxy coatings.

XRD pattern of synthesized nanocomposite coatings containing 5 wt.% TNTs loaded with DOC is shown in Figure 3. A broad peak at 2θ~20° is a characteristic peak of epoxy showing its amorphous polymeric nature. Some other peaks of TiO_2_ are also seen in the pattern due to the use of TNTs as nanocontainers. No peaks of DOC were noticed in the XRD pattern.

### 3.3. FTIR Analysis

**Figure 4:**
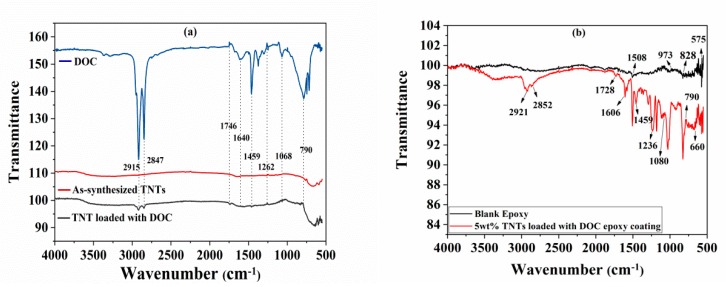
FTIR spectra of (**a**) as-synthesized TNTs, TNTs loaded with DOC, and pure DOC; (**b**) pure epoxy and epoxy coatings containing 5 wt.% of TNTs loaded with DOC.

FTIR spectra of as-synthesized TNTs and TNTs loaded with DOC are shown in Figure 4a, while FTIR spectra of Pure epoxy and as synthesized nanocomposite coatings are shown in Figure 4b. The FTIR analysis of two different spectra in Figure 4a shows that the characteristic band for TNT can be allocated to the bending and stretching vibration for Ti-O-Ti at around 660 cm^−1^ while in case of TNTs loaded with DOC, the first band observed around 3000 cm^−1^ and the peaks at 2915 cm^−1^ and 2847 cm^−1^ correspond to pure DOC which confirms the loading of TNTs with DOC in Figure 4a. The band at 2847 cm^−1^ corresponds to the C–H stretching vibration [39]. The band at 1728 cm^−1^ is due to stretching vibration of C=O. The N–H band is usually observed at about 1650 cm^−1^ [40]. However, in our study, the N–H bending vibration observed at 1640 cm^−1^. This shift in band from its original position exhibits a hydrogen bonding nature while the band at 1459 cm^−1^ is due to C=C stretching vibration. The bands at 1236 cm^−1^ and 1080 cm^−1^ are from –-N bonding and N–H deformation, respectively. The C–H bending vibration can also be observed at 790 cm^−1^. Figure 4b shows the FTIR spectra of blank epoxy and TNT loaded with DOC epoxy coatings. It has been seen that the peak at 1508 cm^−1^ corresponds to the C–C stretching vibrations in aromatic. The peak at 973 corresponds to the characteristic peak of epoxide ring vibrations. The peak at 828 cm^−1^ refers to the C–H plane deformation in aromatic. Moreover, the peak at 575 cm^−1^ corresponds to characteristics frequency of C_6_H_4_X_2_-para (X represents any functional group), which is also in accordance of previous reported literature [41].

### 3.4. Thermal Stability

**Figure 5:**
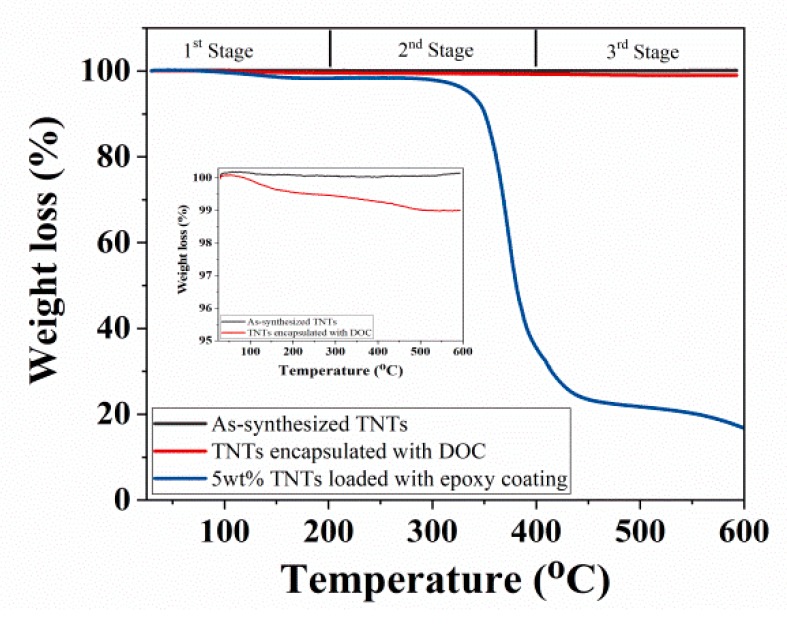
TGA analysis of as-synthesized TNTs, TNTs loaded with DOC, and nanocomposite.

Thermal analysis of as-synthesized TNTs, TNTs loaded with DOC, and developed nanocomposite coatings are compared in Figure 5. It is noticed that the nanocomposite coatings remain quite stable in the first stage from room temperature to about 200 °C. However, there is a significant weight loss of the nanocomposite coatings observed in second stage, when the temperature is increased from 300 °C to 400 °C. This weight loss can be attributed to the thermal degradation of epoxy. There is almost no weight loss observed for the nanocomposite coatings in the third stage (400–600 °C). As a comparison, TNTs remains stable (no weight loss) up to 600 °C (being a ceramic phase). A small amount of weight loss (0.58%) of TNTs is probably due to the removal of moisture content. Similarly, TNTs loaded with DOC shows no weight loss in the first stage but small weight loss observed in the second stage from about 200 °C. A total of 1% weight loss occurred in TNTs encapsulated with DOC and this is probably due to the thermal decomposition of DOC.

### 3.5. UV-vis Spectroscopy

**Figure 6:**
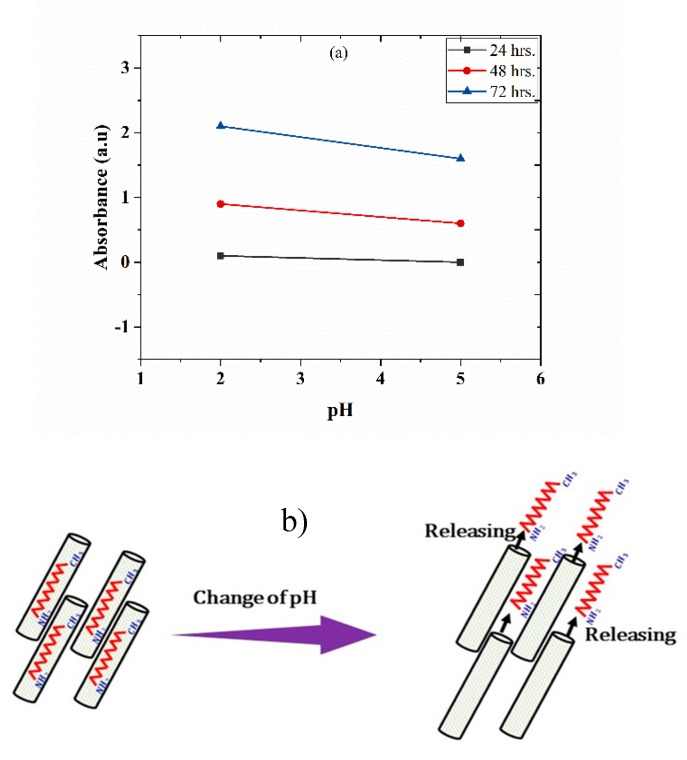
(**a**) UV spectra of TNTs encapsulated with DOC immersed in 0.1M NaCl solution at pH 2 and pH 5 and (**b**) schematic diagram showing the self-release of DOC from TNTs in response to pH change.

The release of DOC inhibitor from TNTs with respect to time at different pH values has been studied by UV-vis spectroscopy. Figure 6a shows the UV-vis spectroscopic analysis of TNTs loaded with DOC immersed into a 0.1 M NaCl solution at pH 2 and 5. Absorbance peaks showing the release of DOC from TNTs in response to pH change can be noticed. The absorbance value at 200 and 230 nm can be assigned to the presence of inhibitor in the solution. There is no significant absorbance value observed in the 24 h, indicating no release of DOC from the TNTs. However, after the 48 h, peak intensity value shows the release of inhibitor in response to pH change. There is an increase in the intensity of peaks after 72 h, showing more release of inhibitor form TNTs. Almost the same peak intensity was observed at both pH 2 and 5 but a slightly higher peak was observed at pH 2 as compared to pH 5. This analysis indicates that the release of DOC from TNTs is time-dependent and sensitive to the aggressiveness of the corrosive medium. Local change in the pH during corrosion process act as the trigger for the release of DOC from TNT as initiation of corrosion activity is accompanied by local change of pH in anodic and cathodic sides [20]. TNTs loaded with DOC perform better in a more acidic environment. This is consistent with the previous studies [27]. The self-release of DOC from TNTs in response to pH change is also schematically shown in Figure 6b.

### 3.6. Zeta Potential Analysis

**Figure 7:**
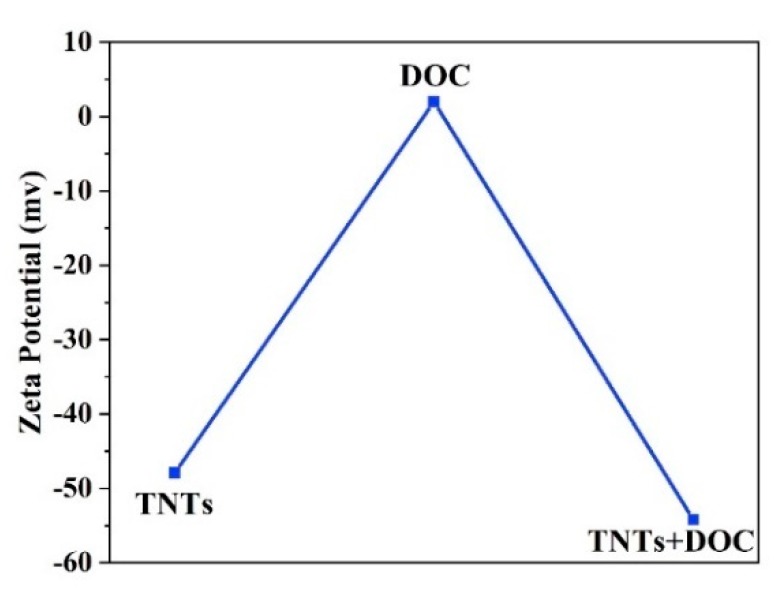
Zeta potential values of TNTs, DOC, and TNTs loaded with DOC.

The potential stability of the colloidal system was studied by zeta potential as a function of the TNTs concentration in deionized water. Figure 7 shows the zeta potential values of TNTs, DOC, and TNTs loaded with DOC. As-synthesized TNTs and TNTs loaded with DOC exhibit negative zeta potential. The zeta potential value for the as-synthesized TNTs is −47.9 mV while DOC has a slightly positive charge of 2 mV. Addition of the DOC into TNTs causes a slight decrease in the value of the zeta potential (−54.2 mV). It should be noted that a zeta potential value greater than 30 mV indicates good stability of the TNTs. This analysis suggests that no agglomeration takes place. Furthermore, the change in the concentration of the loaded TNT with DOC does not affect the zeta potential considerably. This is expected since previous studies have shown that a great shift in zeta potential values are observed for concentrations below 10^−4^ wt.% [42].

### 3.7. Electrochemical Impedance Spectroscopy (EIS)

**Figure 8:**
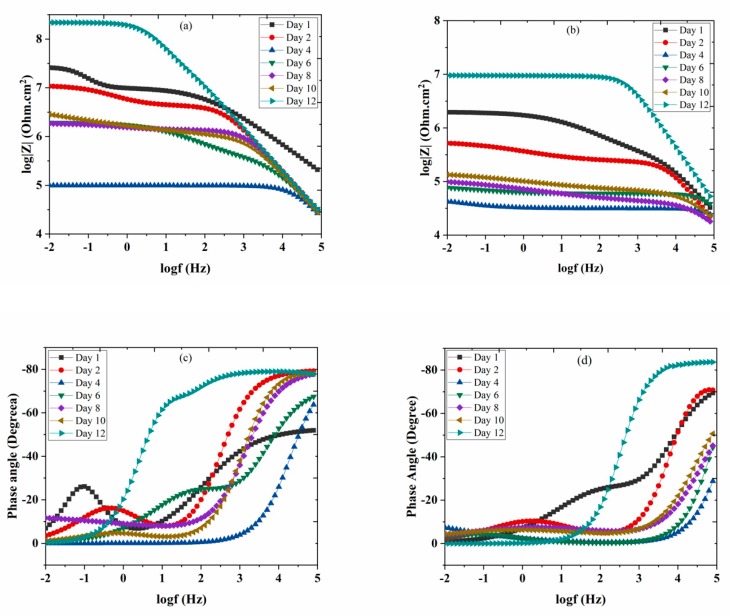

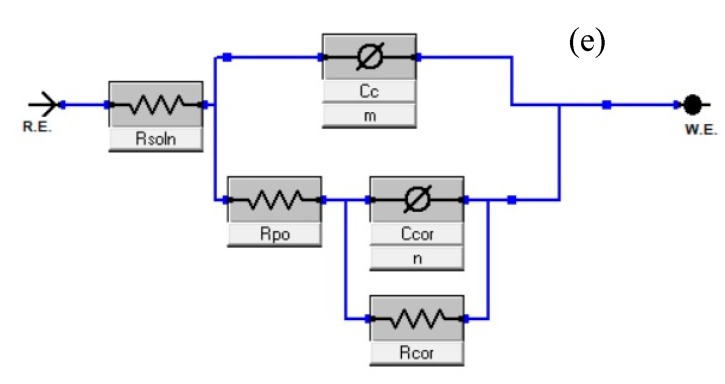
Bode graph of TNTs loaded with DOC (**a**), 5 wt.% at pH 2 (**b**), 5 wt.% at pH 5, phase angle measured of TNTs loaded with DOC (**c**), 5 wt.% at pH 2 (**d**), 5 wt.% at pH 5 (**e**); equivalent circuit used for fitting of EIS data.

**Figure 9:**
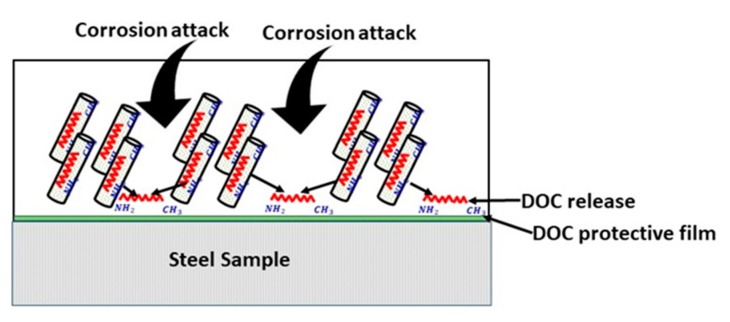
Schematic diagram of the healing mechanism in nanocomposite coatings.

Corrosion resistance behavior of nanocomposite coatings deposited on carbon steel was studied by EIS measurements. The coated samples were immersed in 3.5 wt.% NaCl solution at pH values of 2 and 5. Corrosion resistance of the coated samples was studied up to 12 days of immersion. Figure 8a,b shows the bode plots of nanocomposite coatings at pH 2 and 5. Phase angles measured at pH 2 and pH 5 are also shown in Figure 8c,d. Table 1 shows the electrochemical impedance parameters obtained from EIS analysis at pH 2 and pH 5. The equivalent circuit used for fitting of EIS data is also shown in Figure 8e. At pH 2, the charge transfer resistance (*R*_ct_) after one day of immersion for nanocomposite coatings is measured to be as 16.54 × 10^6^ Ω.cm^2^ with a lower value of phase angle. Higher *R*_ct_ value shows that at the beginning nanocomposite coatings are able to protect themselves from corrosion attack. However, this value dropped down to 136.2 × 10^3^ Ω.cm^2^ after four days of immersion in the solution indicating that the coatings have started losing their ability to resist the corrosive attack. At the later days of immersion, the *R*_ct_ value again shows an increase by reaching a maximum value of 148.2 × 10^6^ Ω.cm^2^ with a major shift in the value of the phase angle (after 12 days of immersion). This increase in the *R*_ct_ value after four days, is due to the release of the DOC from the loaded nanotubes, which is forming a protective film in the scratched area, hence mitigating the corrosion phenomenon in that region. A schematic diagram showing the coatings healing mechanism is shown in Figure 9. The release of DOC from TNTs and the formation of a protective layer of DOC to inhibit corrosion attack is presented to explain the inhibition process.

The same trend has been observed during the EIS analysis of coated samples at pH 5. After one day of immersion, the *R*_ct_ value of the coated sample was measured to be 2.651 × 10^6^ Ω.cm^2^ while the *C*_c_ value was about 423.2 x10^-12^ F/cm^2^ with a lower value of measured phase angle. The higher *R*_ct_ and lower *C*_c_ value indicate good resistance of coatings against the corrosive attack. As the immersion time of the sample increases, the *R*_ct_ value at pH 5 shows a decreasing trend until the fourth day of immersion. This decrease indicates that the release of DOC from the nanocontainers has not yet started and hence the coating has started to lose its protective ability. The corrosion resistance of the coating starts to increase after day 6 due to the release of the inhibitor. However, at day 10 and day 12, *R*_ct_ value reaches to 91.31 × 10^3^ Ω.cm^2^ and 7.683 × 10^6^ Ω.cm^2^, respectively. With the increasing *R*_ct_ value, a major shift in the value of the phase angle has also been observed. This increase in the *R*_ct_ value indicates that the self-release of DOC has started from TNTs and, thus, it protects the coating surface from the corrosion attack by forming a protective layer over the surface. These observations suggest that the release of the inhibitor (DOC) from TNTs is time-dependent. Similar behavior was also confirmed by the displacement of phase angle from higher frequencies to lower frequencies in the bode diagram (θ vs. log f). This trend demonstrates the further protective role of DOC released from the TNTs particles over the passage of time, which results in forming an adsorbed film on the surface of carbon steel. For the coated samples containing the same weight percentages of loaded nanocontainers, tested at pH 2 and pH 5, rendering to the results of releasing kinetics of releasing of DOC at different pH conditions, it can be concluded that for pH 2.0, there is a greater amount of released inhibitor from the encapsulated TNTs. Hence, superior anticorrosive properties at pH 2 are obtained when compared with pH 5. These properties and releasing kinetics of the coatings make them attractive and are promising for the subsequent application of the loaded TNTs in anticorrosive smart coatings, as the corrosion is usually followed by alkaline or acidic pH shift. Thus, a release of the inhibitor in response to a pH change in the local surrounding environment occurs which can protect the steel substrate in the damaged area of the coating. A comparison of the present study with the already reported systems has been tabulated in Table 2, which demonstrates the efficiency and better anti-corrosive properties of the present study in comparison to the already reported similar systems.

For the coated samples containing the same weight percentages of loaded nanocontainers, tested at pH 2 and pH 5, EIS parameters confirm that the developed nanocomposite coatings demonstrate superior corrosion resistance at pH 2 when compared with pH 5. The time-dependent release of DOC from the nanocontainers is more effective at pH 2. Therefore, this system is sensitive to the pH shift in the acidic region releasing the inhibitor on demand when the anodic process starts. Hence, more of the inhibitor is released from the nanocontainer in less time at pH 2 as compared to pH 5 [43]. This analysis shows that the release of DOC from TNTs is sensitive to the pH of the corrosive medium, which is consistent with previous studies [44]. This section may be divided by subheadings. It should provide a concise and precise description of the experimental results, their interpretation, as well as the experimental conclusions that can be drawn.

## 4. Conclusions

Polymeric nanocomposite coatings were developed by reinforcing epoxy matrix with TNTs loaded with DOC and their performance was evaluated in corrosive environments. Structural, morphological, and thermal analysis results confirm the successful loading of DOC into TNTs. It was also confirmed that the self-release of DOC from the TNTs is time and pH sensitive. The developed nanocomposite coatings demonstrate superior anticorrosion properties at pH 2 when compared to pH 5. The improved electrochemical performance of nanocomposite coatings can be attributed to the excessive and more effective release of DOC from the TNTs at pH 2. The tempting anticorrosion properties of synthesized polymeric nanocomposite coatings in the acidic environment make them attractive for industrial applications.

## Figures and Tables

**Table 1:** Electrochemical impedance parameters from measured impedance data for coating of 5 wt.% TNTs loaded with DOC at pH 2 and 5.

Coating	Time (Day)	*R*_ct_ (Ω·cm^2^)	*C*_C_ (F/cm^2^)	*R*_PO_ (Ω·cm^2^)	*C*cor (F/cm^2^)
5 wt.% TNTs loaded with DOC at pH 2	1	16.54 × 10^6^	2.128 × 10^−9^	10.11 × 10^6^	166.4 × 10^−9^
	2	6.914 × 10^6^	287.8 × 10^−12^	4.284 × 10^6^	117.0 × 10^−9^
	4	136.2 × 10^3^	319.5 × 10^−12^	98.79 × 10^3^	101.9 × 10^−6^
	6	1.561 × 10^6^	811.1 × 10^−12^	378.5 × 10^3^	50.68 × 10^−9^
	8	28.79 × 10^6^	325.4 × 10^−12^	801.7 × 10^3^	763.4 × 10^−9^
	10	520.0 × 10^3^	252.2 × 10^−12^	1.355 × 10^6^	1.005 × 10^−6^
	12	148.2 × 10^6^	301.1 × 10^−12^	69.95 × 10^6^	110.1 × 10^−12^
5 wt.% TNTs loaded with DOC at pH 5	1	2.651 × 10^6^	423.2 × 10^−12^	265.4 × 10^3^	34.41 × 10^−9^
	2	328.9 × 10^3^	289.6 × 10^−12^	231.9 × 10^3^	1.986 × 10^−6^
	4	11.56 × 10^3^	445.2 × 10^−12^	32.01 × 10^3^	278.8 × 10^−6^
	6	23.11 × 10^3^	76.82 × 10^−12^	59.49 × 10^3^	77.51 × 10^−6^
	8	76.61 × 10^3^	1.678 × 10^−9^	39.52 × 10^3^	10.13 × 10^−6^
	10	91.31 × 10^3^	1.624 × 10^−9^	65.25 × 10^3^	9.279 × 10^−6^
	12	7.683 × 10^6^	66.88 × 10^−12^	1.798 × 10^6^	699.21 × 10^−12^

**Table 2:** Electrochemical impedance parameters from measured impedance data for coating of 5 wt.% TNTs loaded with DOC at pH 2 and 6.2.

Sr. No	Coatings	Immersion time	*R*_ct_	References
1	1 wt.% of nanocontainers (MS) with encapsulated DOC at pH 2	1 hr.	5.00 × 10^2^ Ω·cm^2^	[43]
		3 hrs.	6.50 × 10^2^ Ω·cm^2^	
		5 hrs.	7.50 × 10^2^ Ω·cm^2^	
		16 hrs.	2.500 × 10^3^ Ω·cm^2^	
	1 wt.% of nanocontainers (MS) with encapsulated DOC at pH 6.2	1 hr.	1.500 × 10^3^ Ω·cm^2^	
		3 hrs.	1.750 × 10^3^ Ω·cm^2^	
		5 hrs.	2.000 × 10^3^ Ω·cm^2^	
		16 hrs.	3.000 × 10^3^ Ω·cm^2^	
2	1 wt.% Halloysite nanotubes with encapsulated DOC at pH 2	1,2,3,5 and 7 hrs.	2.00 × 10^2^ Ω·cm^2^	[45]
		18 hrs.	5.50 × 10^2^ Ω·cm^2^	
		36 hrs.	8.00 × 10^2^ Ω·cm^2^	
		48 hrs.	1.100 × 10^3^ Ω·cm^2^	
	1 wt.% Halloysite nanotubes with encapsulated DOC at pH 6.2	1,2,3,5 and 7 hrs.	1.700 × 10^3^ Ω·cm^2^	
		18,36 and 48 hrs.	2.000 × 10^3^ Ω·cm^2^	
3	1 wt.% of highly ordered Mesoporous silica with encapsulated DOC at pH 2	1,2,3,5 and 6 hrs.	7.50 × 10^2^ Ω·cm^2^	[28]
		24, 26, 28 hrs.	1.750 × 10^3^ Ω·cm^2^	
	1 wt.% of highly ordered Mesoporous silica with encapsulated DOC at pH 6.2	1,2,3,5 and 6 hrs.	2.250 × 10^3^ Ω·cm^2^	
		24, 26, 28 hrs.	3.250 × 10^3^ Ω·cm^2^	
